# Genotyping of *Listeria monocytogenes* isolates from poultry carcasses using high resolution melting (HRM) analysis

**DOI:** 10.1080/13102818.2014.901681

**Published:** 2014-05-06

**Authors:** Ioannis Sakaridis, Ioannis Ganopoulos, Panagiotis Madesis, Athanasios Tsaftaris, Anagnostis Argiriou

**Affiliations:** ^a^ Institute of Applied Biosciences, Centre for Research and Technology Hellas, Thessaloniki, Greece

**Keywords:** HRM, *L. monocytogenes*, RAPD, poultry carcasses, genotyping

## Abstract

An outbreak situation of human listeriosis requires a fast and accurate protocol for typing *Listeria monocytogenes*. Existing techniques are either characterized by low discriminatory power or are laborious and require several days to give a final result. Polymerase chain reaction (PCR) coupled with high resolution melting (HRM) analysis was investigated in this study as an alternative tool for a rapid and precise genotyping of *L. monocytogenes* isolates. Fifty-five isolates of *L. monocytogenes* isolated from poultry carcasses and the environment of four slaughterhouses were typed by HRM analysis using two specific markers, *internalin B* and *ssrA* genes. The analysis of genotype confidence percentage of *L. monocytogenes* isolates produced by HRM analysis generated dendrograms with two major groups and several subgroups. Furthermore, the analysis of the HRM curves revealed that all *L. monocytogenes* isolates could easily be distinguished. In conclusion, HRM was proven to be a fast and powerful tool for genotyping isolates of *L. monocytogenes*.

## Introduction

*Listeria monocytogenes* is a Gram positive, motile, non-sporulating bacterium and is the causative agent of listeriosis in both humans and animals. It is widely distributed in the environment and has the ability to survive and grow under extreme conditions, like low temperature and high salt levels. Several studies have proved that *L. monocytogenes* is capable of causing encephalitis, meningitis and septicemia and is also accounted for a number of food-borne outbreaks of listeriosis.[1,2] Unlike many other bacterial diseases associated with food, listeriosis presents a high fatality rate (10%–30%),[3] especially among high risk population groups. Pregnant women, neonates, adults with underlying disease, the elderly (>65) and other immunocompromised individuals are particularly susceptible to infection.[4] Thus, *L. monocytogenes* has been recognized as an emerging food-borne pathogen and has become a major concern to the food industry and to the general public over the last few decades.

When an outbreak situation occurs, a fast and accurate protocol for subtyping *L. monocytogenes* is necessary. There are several serotyping and molecular based methods for conducting epidemiological tracing of specific isolates of *L. monocytogenes*. The Listeria serotyping scheme based on somatic (O) and flagellar (H) antigens currently represents a standard for *L. monocytogenes* isolate typing and investigations into the ecological distribution, epidemiology and virulence of isolates. Unfortunately, serotyping discriminates only 13 serotypes, many of which are known to represent genetically diverse groups of isolates, yet only four serotypes (1/2a, 1/2b, 1/2c, and 4b) cause almost all cases of listeriosis in humans. Moreover, serotyping based schemes have limited value for tracking isolates since they are characterized by insufficient reproducibility, relatively low discriminatory power and antigen sharing among serotypes.[5] Therefore, there is a need for more accurate and fast molecular based typing methods.

Random amplification of polymorphic DNA (RAPD) and pulse-field gel electrophoresis (PFGE) are two of the most common molecular based methods for typing. RAPD is a fast and simple molecular typing method and although it is characterized by inadequate intra- and interlaboratory reproducibility, the intralaboratory variation can be minimized by standardization of DNA extraction and PCR conditions.[6] The other method, PFGE, is the current gold standard for typing *L. monocytogenes* isolates, even though it is time consuming and difficult to standardize, which hampers interlaboratory exchange and comparison of typing results.[7]

Alternative typing methods, based on DNA sequence analysis and single nucleotide polymorphism (SNP) detection could be introduced for a fast and accurate strain typing assay, especially during an outbreak when a fast and accurate method is needed in order to ensure public health. As stated by Pietzka et al.,[8] a PCR-based typing method targeting a single genetic region would be superior considering the cost, ease, turnaround time, and potential for standardization for a rapid identification and typing of isolates in routine diagnostics. High resolution melting (HRM) analysis is a closed tube, post-PCR, DNA based method that is applicable for genotyping and fingerprinting by distinguishing DNA sequence variants based on the shape of melting transitions (Tm) of real-time PCR products.[9,10] It is considered a rapid and precise method with high-throughput possibilities, which is simpler and less expensive than alternative methods requiring post-PCR processing, enzyme restriction and electrophoresis, or labelled probes for SNP detection sequencing or TaqMan-probe-based real-time PCR.[11] In addition, it is suggested that its specificity is superior when compared to probe-dependent classical PCR genotyping methods and is comparable to DNA sequencing.[8] Two specific genes have been selected for the genotyping assay of the *L. monocytogenes* isolates. The first one was the *internalin B* gene that has been used in a study by Pietzka et al. [8] because it was the one with the highest genetic variability. The second one was the *ssrA* gene that was successfully applied [12] for the molecular identification of *Listeria* species.

The objective of the present study was to apply and validate the use of HRM for the genotyping of 55 *L. monocytogenes* isolates, by discriminating the DNA sequences variations of the *internalin B* and *ssrA* genes. A distinct HRM assay was applied for each gene.

## Materials and methods

Fifty-five samples of *L. monocytogenes* isolates (Table 1) were obtained from poultry carcasses and the environment of four slaughterhouses in a previous study.[13] The isolates were stored at −80 ºC in microbanks (PRO-LAB Diagnostics, Richmond Hill, ON, Canada) until use. The selected isolates were cultivated at 37 ºC in Tryptic Soy Yeast Extract agar for 48 h and then recultivated for another 48 h. DNA was extracted from these isolates using Nucleospin Tissue kit (Macherey Nagel, Duren, Germany) following the instructions given by the manufacturer.

**Table 1.** *L. monocytogenes* isolates used in this study.

Number of isolates	*L. monocytogenes* strain	Origin
1	10	Poultry carcasses
2	11	Poultry carcasses
3	12	Poultry carcasses
4	207	Poultry carcasses
5	208	Poultry carcasses
6	215	Poultry carcasses
7	216	Poultry carcasses
8	302	Poultry carcasses
9	303	Poultry carcasses
10	304	Poultry carcasses
11	305	Poultry carcasses
12	403	Poultry carcasses
13	404	Poultry carcasses
14	501	Poultry carcasses
15	502	Poultry carcasses
16	504	Poultry carcasses
17	505	Poultry carcasses
18	510	Poultry carcasses
19	512	Poultry carcasses
20	513	Poultry carcasses
21	514	Poultry carcasses
22	515	Poultry carcasses
23	801	Poultry carcasses
24	802	Poultry carcasses
25	803	Poultry carcasses
26	804	Poultry carcasses
27	805	Poultry carcasses
28	806	Poultry carcasses
29	807	Poultry carcasses
30	808	Poultry carcasses
31	809	Poultry carcasses
32	810	Poultry carcasses
33	903	Poultry carcasses
34	904	Poultry carcasses
35	905	Poultry carcasses
36	906	Poultry carcasses
37	908	Poultry carcasses
38	909	Poultry carcasses
39	911	Poultry carcasses
40	912	Poultry carcasses
41	913	Poultry carcasses
42	914	Poultry carcasses
43	920	Poultry carcasses
44	921	Poultry carcasses
45	922	Poultry carcasses
46	923	Poultry carcasses
47	925	Poultry carcasses
48	931	Refrigerator door handles
49	932	Refrigerator door handles
50	933	Containers with chickens
51	934	Containers with chickens
52	936	Containers without chickens
53	938	Work surfaces
54	939	Cutting boards
55	940	Cutting boards

The *internalin B* and *ssrA* genes from these DNA samples were amplified using primers that annealed to conserved regions of the genes. These primers were the forward *inlB* 5′-CAT GGG AGA GTA ACC CAA CC-3′ and reverse *inlB* 5′-GCG GTA ACC CCT TTG TCA TA-3′ [8] and the forward *ssrA* 5′-CGT GCA TCG CCC ATG TGC-3′ and reverse *ssrA* 5′-ATC TAC GAG CGT AGT CAC-3′,[12] respectively.

PCR amplification, DNA melting and end point fluorescence level acquiring PCR amplifications were performed in a total volume of 10 μL on a Rotor-GeneQ real-time 5Plex HRM PCR Thermocycler (QIAGEN GmbH, Mannheim, Germany) according to Pietzka et al. [8] and Jin et al. [12].

A rapid PCR protocol and HRM analysis were conducted in a 72-well carousel using an initial denaturing step of 95 °C for 3 min followed by 40 cycles of 95 °C for 20 sec, 60 °C for 30 sec and 72 °C for 40 sec, and then a final extension step of 72 °C for 2 min. Before HRM, the products were denatured at 95 °C for 5 sec, and then annealed at 50 °C for 30 sec to randomly form DNA duplexes. HRM was performed as follows: pre-melt at the first appropriate temperature for 90 sec, and melt at a ramp of 10 °C in an appropriate temperature range at 0.1 °C increments every 2 sec. The fluorescent data were acquired at the end of each annealing step during PCR cycles. End point fluorescence level was acquired following the melting process by holding at 60 °C for 5 min and five cycles of 60 °C for 20 sec with fluorescence data being acquired at the end of each cycle step. PCR products were analysed on a 2% agarose gel in order to ensure the amplification of the correct size products (data not shown). All experiments were performed in triplicate measurements.

The resulting melting profiles were analysed by the XLSTAT version 2012 software (http://www.xlstat.com). The similarity among genotype confidence profiles was calculated using the Pearson correlation, and an average linkage (UPGMA – unweighted pair group method with arithmetic mean) dendrogram was derived from the profiles.

## Results and discussion

**Figure 1.**
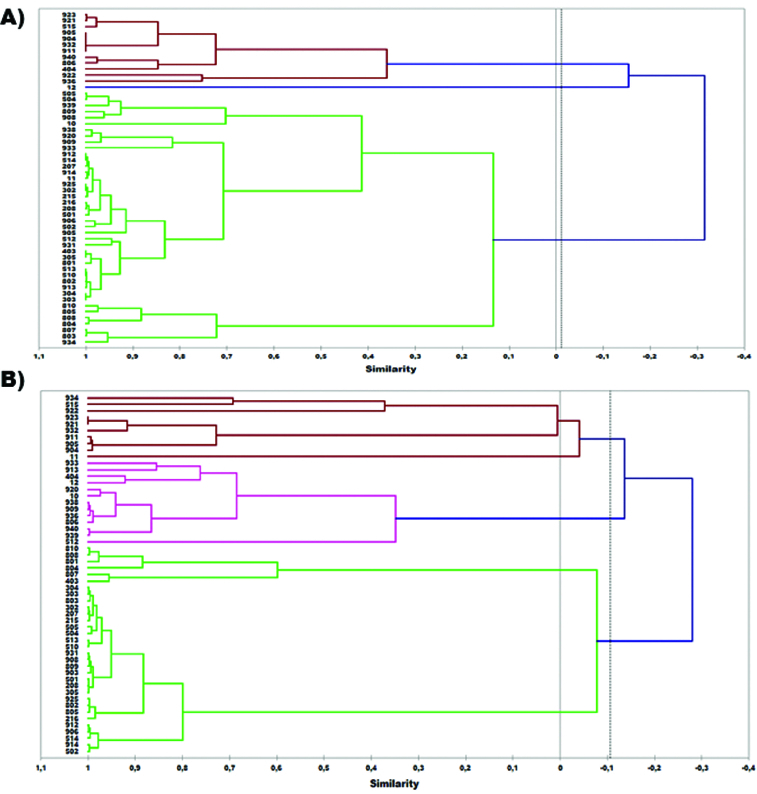
Dendrogram of 55 *L. monocytogenes* isolates based on UPGMA analysis of: (A) *inlB* and (B) *ssrA* markers.

Both *inlB* and *ssrA* genes were selected for genotyping the 55 isolates of *L. monocytogenes* and for evaluating the applicability of HRM analysis to discriminate these isolates. First, DNA from the 55 *L. monocytogenes* isolates that originated from poultry carcasses was genotyped via HRM using the approximately 500 bp (base pair) products from the *inlB* gene that was amplified by the specific primer pair. Figure 1(A) depicts the dendrogram produced by the genotype confidence percentage (GCP) of isolates obtained by HRM analysis. All *L. monocytogenes* isolates were allocated in two major groups (A and B). Thirteen isolates were found to belong to the first group and 42 to the second. Taking into account that isolates presenting similarity more than 80% in their melting profiles were considered to belong to the same subgroup, five distinct subgroups were obtained in group A and six in group B. The most populated subgroup was B_4_ containing 25 isolates, while all other subgroups consisted of fewer isolates (1–8).

A 160 bp product from the *ssrA* gene was also amplified using the specific primer pair and DNA extracted from the 55 isolates. The dendrogram produced by the GCPs of isolates obtained by HRM analysis is presented in Figure 1(B). All *L. monocytogenes* isolates were also allocated in two distinct groups (A and B). Group B contained 32 isolates and subgroup B_3_ was the most numerous, consisting of 26 isolates.

**Figure 2.**
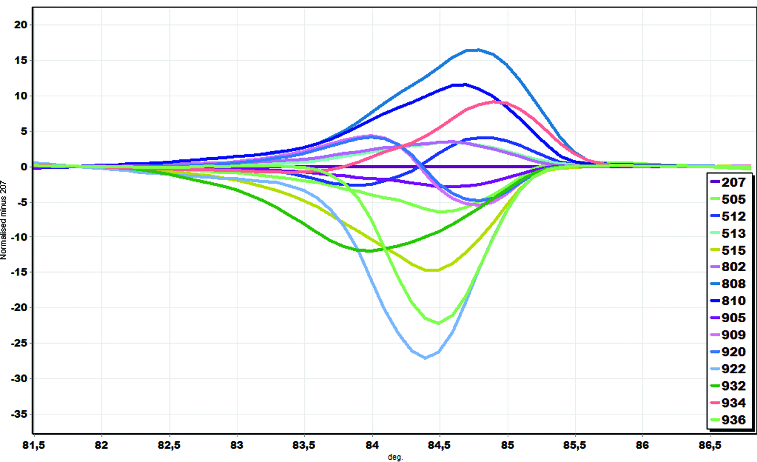
Melting curve analysis of *inlB* marker in a representative set of *L. monocytogenes* isolates. HRM differential plot using ‘strain 207’ as reference.

Analysis of the normalized HRM curves produced with the *inlB* marker revealed that all isolates could easily be distinguished. Furthermore, closer examination of the *L. monocytogenes* curves, with the curve of strain 207 as the baseline, revealed part of the curve sitting outside the 80% CI (confidence interval) curve, suggesting that a significant number of examined *L. monocytogenes* isolates via the HRM curves are indeed different (Figure 2). Arbitrarily assigning the strain 207 as a genotype, we were able to estimate the confidence value of similarity between 207 and the other *L. monocytogenes* isolates used in the study and to show that *inlB* was a sufficient region to distinguish the tested isolates. GCPs were calculated, and a cut-off value of 80% was used to assign a genotype for each region. Similar results were obtained with *ssrA* marker (data not shown).

Comparing the dendrograms produced by *inlB* and *ssrA* genes it can be concluded that there are many resemblances concerning the genotyping of the *L. monocytogenes* isolates. Both dendrograms contained two major groups and several isolates were allocated to the same groups. Similarities were also observed among subgroups of the two dendrograms. To be more precise, 19 isolates of *L. monocytogenes* were found to be common among the subgroup B_4_ of *inlB* and B_3_ of the *ssrA* dendrogram. Differences in the dendrograms correspond to the degree of variance in the DNA sequence of the two genes among the *L. monocytogenes* isolates. Nevertheless, both dendrograms present a high capability of distinguishing those isolates.

The dendrograms produced by HRM analysis were also compared to the dendrogram produced by RAPD for the same isolates of *L. monocytogenes* in a previous study.[13] There is an obvious resemblance among all three dendrograms and especially for the subclade A1 of the RAPD dendrogram which is similar to the above-mentioned B_4_ (*inlB*) and B_3_ (*ssrA*) subgroups. However, the dendrograms produced by HRM analysis data present a higher resolution in separating the different *L. monocytogenes* isolates valourizing this method as an alternative to RAPD analysis.

HRM analysis has also been successfully applied for identifying and distinguishing *Fusarium oxysporum* formae speciales complex and generated seven HRM curve profiles resulting in the classification of the isolates into seven *F. oxysporum* formae speciales.[14] Recently, its use for genotyping food-borne bacteria is under investigation and a few assays concerning the genotyping of pathogenic micro-organisms have been published.[8,12,15,16]

## Conclusions

Overall, HRM is a cost-effective and high-throughput tool for amplification and genotyping the *L. monocytogenes* isolates. It requires approximately 1 h per run including the follow-up data analysis. Thus, this method is a suitable tool for fast and accurate genotyping of *L. monocytogenes* isolates.

## References

[cit0001] Liu D, Ainsworth AJ, Austin FW, Lawrence ML (2003). J Med Microbiol.

[cit0002] Werbrouck H, Botteldoorn N, Uyttendaele M, Herman L, Van Coillie E (2007). J Microbiol Methods.

[cit0003] Doganay M (2003). FEMS Immunol Med Microbiol.

[cit0004] Ceylan ZG, Demirkaya AK, Adiguzel G (2008). J Food Quality.

[cit0005] Liu D (2006). J Med Microbiol.

[cit0006] Gravesen A, Jacobsen T, Moller PL, Hansen F, Larsen AG, Knochel S (2000). Int J Food Microbiol.

[cit0007] Kabuki DY, Kuaye AY, Wiedmann M, Boor KJ (2004). J Dairy Sci.

[cit0008] Pietzka AT, Stoger A, Huhulescu S, Allerberger F, Ruppitsch W (2011). J Mol Diagn.

[cit0009] Wittwer CT (2009). Hum Mutation.

[cit0010] Zhou L, Wang L, Palais R, Pryor R, Wittwer CT (2005). Clin Chem.

[cit0011] Reed GH, Kent JO, Wittwer CT (2007). Pharmacogenomics.

[cit0012] Jin D, Luo Y, Zhang Z, Fang W, Ye J, Wu F, Ding G (2012). FEMS Microbiol Lett.

[cit0013] Sakaridis I, Soultos N, Iossifidou E, Papa A, Ambrosiadis I, Koidis P (2011). J Food Protect.

[cit0014] Ganopoulos I, Madesis P, Zambounis A, Tsaftaris A (2012). FEMS Microbiol Lett.

[cit0015] Levesque S, Michaud S, Arbeit RD, Frost EH (2011). PLoS One.

[cit0016] Robertson T, Bibby S, O’Rourke D, Belfiore T, Lambie H, Noormohammadi AH (2009). J Appl Microbiol.

